# Combined xanthorrhizol-curcumin exhibits synergistic growth inhibitory activity *via *apoptosis induction in human breast cancer cells MDA-MB-231

**DOI:** 10.1186/1475-2867-9-1

**Published:** 2009-01-02

**Authors:** Yew Hoong Cheah, Fariza Juliana Nordin, Rozie Sarip, Thiam Tsui Tee, Hawariah Lope Pihie Azimahtol, Hasnah M Sirat, Badrul Amini Abd Rashid, Noor Rain Abdullah, Zakiah Ismail

**Affiliations:** 1 Bioassay Unit, Herbal Medicine Research Center, Institute for Medical Research, Jalan Pahang, Kuala Lumpur, Malaysia; 2 School of Biosciences and Biotechnology, Faculty of Science and Technology, National University of Malaysia, 43600 UKM Bangi, Selangor, Malaysia; 3 Department of Chemistry, Faculty of Science, Universiti Teknologi Malaysia, 80310 Skudai, Johor, Malaysia; 4 Phytochemistry Unit, Herbal Medicine Research Center, Institute for Medical Research, Jalan Pahang, Kuala Lumpur, Malaysia

## Abstract

**Background:**

It has been suggested that combined effect of natural products may improve the treatment effectiveness in combating proliferation of cancer cells. The present study was undertaken to evaluate the possibility that the combination of xanthorrhizol and curcumin might show synergistic growth inhibitory effect towards MDA-MB-231 human breast cancer cells *via *apoptosis induction. The effective dose that produced 50% growth inhibition (GI_50_) was calculated from the log dose-response curve of fixed-combinations of xanthorrhizol and curcumin generated from the sulforhodamine B (SRB) assay. The experimental GI_50 _value was used to determine the synergistic activity of the combination treatment by isobolographic analysis and combination-index method. Further investigation of mode of cell death induced by the combination treatment was conducted in the present study.

**Results:**

Isobole analysis revealed that substances interaction was synergistic when xanthorrhizol and curcumin were added concurrently to the cultures but merely additive when they were added sequentially. The synergistic combination treatment was then applied to the cultures to investigate the mode of cell death induced by the treatment. Immunofluorescence staining using antibody MitoCapture™ revealed the possibility of altered mitochondrial transmembrane potential, which is one of the hallmark of apoptosis. Hoechst 33258 nuclear staining assay showed the rate of apoptosis of MDA-MB-231 cells to increase in response to the treatment. Apoptotic cell death was further confirmed by DNA fragmentation assay, where internucleosomal excision of DNA was induced upon treatment with xanthorrhizol-curcumin.

**Conclusion:**

This is the first time the combined cytotoxic effect of xanthorrhizol and curcumin on MDA-MB-231 cells has been documented and our findings provide experimental support to the hypothesis that combined xanthorrhizol-curcumin showed synergistic growth inhibitory activity on MDA-MB-231 cells *via *apoptosis induction.

## Background

Drug discovery from medicinal plants continues to provide an important source of new drug leads [1]. Previous studies have reported the ability of xanthorrhizol and curcumin in killing various cancer cell lines [2-7]. Our previous report on the cytotoxicity of xanthorrhizol on human breast carcinoma MCF-7 has proved that xanthorrhizol was able to induced apoptotic cell death [3]. From the study, we suggested that xanthorrhizol may be able to disrupt the cell plasma membrane as the late apoptotic event was happened in a huge magnitude during a short exposure time according to the flow cytometry analysis [3]. The ability of an antiproliferative agent to disrupt the cell plasma membrane was reported to have involved in the cell protein membrane redox homeostasis and thus, facilitates the induction of apoptosis event [8]. Most recently, curcumin was discovered to have membrane-thinning effect and it also weaken the elasticity moduli of 1,2-dioleoyl-sn-glycero-3-phosphocoline (DOPC) bilayer [9]. It is believed that enhanced cytotoxicity of anticancer drugs could result from changes in the biophysical properties and functions of tumor cell membranes induced by the co-substance as suggested in other reports [10-12]. Meanwhile, in a study on antibacterial properties of xanthorrhizol, researchers have suggested the hydrophobic chain of xanthorrhizol structure can penetrate and breakdown the dental plaque biofilm, resulting the death of the microorganism [13,14].

Interestingly, curcumin was reported to possess inhibitory effect on the generation of reactive oxygen spesies (ROS) and the inactivation of c-Jun NH_2_-terminal kinase (JNK) pathway, which has been associated with chemotherapy-mediated induction of apoptosis in tumor cells [15,16]. This was further proven in a study where curcumin was tested to be able to compromise the proapoptotic activity of camptothecin, alkylating agents and anthracyclines. Besides, it decreased the camptothecin- and mechlorethamine-induced mitochondrial cytochrome *c *release and lowering the effectiveness antitumor activity of cyclophosphamide [17]. In some other studies, curcumin instead showed synergistic growth inhibition and stimulate apoptosis on some cancer cells when combined with chemotherapeutic drugs such as bortezomib and 5-fluorouracil [18,19]. Therefore, the combined effect of the substances is more likely depends on the drug-drug interaction. Xanthorrhizol also have the ability to attenuate the activation of JNK in cisplatin-induced hepatoxcity in mice [20]. Although both curcumin and xanthorrhizol inactivated JNK pathway in some studies but apoptotic cell death still likely to occur as curcumin and xanthorrhizol were tested to have alternative pathway in inducing apoptotic cell death such as the p53-dependent pathway [2,3,21] and mitochondrial-dependent pathway [4,22]. Taken together the ability of both curcumin and xanthorrhizol to disrupt the membrane fluidity and inducing apoptotic cell death, we considered the possibility that combined xanthorrhizol-curcumin might synergistically inhibit the growth of cancer cells *via *apoptosis induction.

In the present study, the combined effect of xanthorrhizol and curcumin was tested on highly invasive human breast cancer cells, MDA-MB-231. This cell line is a good candidate for the study of invasive cancer *in vitro*. The MDA-MB-231 cell line has lost its normal breast cell phenotype. The MDA-MB-231 cell line expresses mutant tumor suppressor gene, *p53 *and invasion factors such as MMP-1, MMP-2, MMP-9 and vimentin but they do not expressed factors such as E-cadherin or steroid receptors such as estrogens and progesterone as compared to the other breast cancer cell lines [23-25]. Furthermore, the MDA-MB-231 cell line achieves high tumorigenicity in *nu*/SCID mice and exhibits invasive properties *in vitro *[26]. Therefore, the altered phenotype in MDA-MB-231 cell line is associated with tumor progression, metastases formation and resistance to programmed cell death [27,28]. Hence, any antiproliferative agent that can halt their proliferation is definitely beneficial for the prevention and treatment of invasive cancer.

In order to determine the best combination of xanthorrizol with curcumin, a fixed ratio concentration treatment and different schedules of administration of the test agents were compared to elucidate whether there was schedule dependency. Both xanthorrhizol and curcumin cytotoxicity on MDA-MB-231 cells were determined earlier (data not shown). The interactions between xanthorrhizol and curcumin were evaluated for synergism, additivity, or antagonism using both isobologram and combination index method as preclinical screening test. To investigate the mode of cell death induced by the combined effect of xanthorrhizol and curcumin, we examined whether apoptosis or necrosis cell death was involved.

## Results

### Xanthorrhizol was isolated from *curcuma xanthorrhizza*

**Figure 1:**
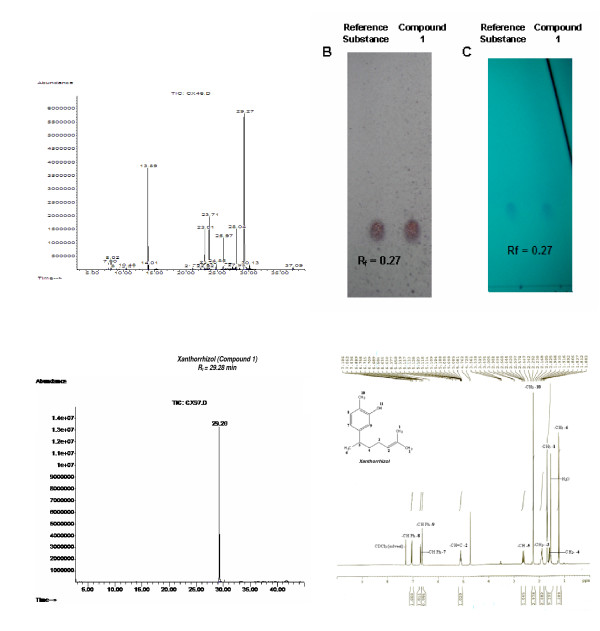
**The extraction and isolation of xanthorrhizol from its crude essential oil**. The designated peak (xanthorrhizol) is the most abundance component, which accounted for 46.3% of the total component in the extracted essential oil of *C. xanthorrhizza *(A). Xanthorrhizol was detected *via *chromogenic reagent spray (0.5% vanillin/sulfuric acid) and UV (254 nm) visualization in TLC profile as compare to the authentic reference substance (B and C). Final isolation process produced compound **1 **at 100% abundance (D) and the NMR spectral identified the compound **1 **as xanthorrhizol (E).

**Figure 2:**
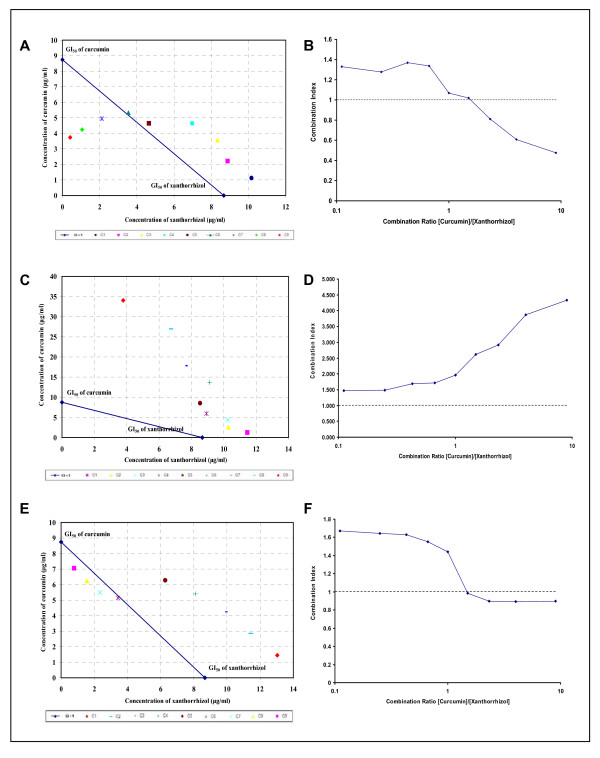
**Simultaneous treatment of xanthorrhizol and curcumin alters the mitochondrial transmembrane potential and induces apoptosis in MDA-MB-231 cells**. MitoCapture™ dye was found localized and fluoresces red (arrow) in the mitochondrial of untreated cells. Number of cells fluoresces red was decreasing in line with the increasing dose of treatment, suggesting the disruption of the mitochondrial transmembrane potential (A). Phase contrast fluorescence images show that untreated cells were stained homogenously and no fluorescence was observed. In contrary, cell shrinkage was observed in the treated cells and fluorescence (arrow) was clearly detected in the nuclear region, indicating apoptotic morphology (B). X – xanthorrhizol. XC – combination of xanthorrhizol and curcumin. TAM – Tamoxifen (positive control).

Hydrodistillation of the freshly chopped rhizome of *Curcuma xanthorrhizza *Roxb. yielded 0.44% of essential oil. GC-MS results showed that xanthorrhizol is the major chemical constituent in the essential oil as it makes up nearly 46.3% of the total component (Figure 1A). Compound **1 **was obtained as light brownish oil and was given name as xanthorrhizol. TLC profile of **1 **presented the R_f _value of 0.27 using 5% diethyl ether in petroleum ether (Figure 1B, 1C). Meanwhile, TIC profile showed an integrated single peak of **1 **with 100% peak area (CAS# 30199-26-9, Figure 1D). Its molecular formula was obtained from high resolution mass spectrum which showed [M^+^] ion at m/z 218.3210 corresponding to the formula C_15_H_22_O; calc 218.3404 (Figure 2). In ^1^H NMR analysis, the presence of phenyl group was evident from signals at *δ*(ppm) 6.63 (s, 1H), 6.71 (dd, 1H, *J *= 7.5, 1.8, 1.5 Hz) and 7.05 (d, 1H, *J *= 7.8 Hz) in the ^1^H NMR spectrum (Figure 1E).

Compound **1 **(xanthorrhizol): Light brownish oil; C15H22O; High MS m/z: M+ 218.3210 calc. 218.3404 for C_15_H_22_O; Low MS m/z (%): 218(42), 48 (28), 136 (100), 121 (57), 135 (54); 1H NMR (300 MHz, CDCl_3_): δ 1.22 (d, 3H, *J *= 6.9 Hz, -CH_3_-**6**), 1.54–1.63 (m, 2H, -CH_2_-**4**), 1.70 (s, 6H, -C(CH_3_)_2_-**1**), 1.87–1.94 (m, 2H, -CH_2_-**3**), 2.24 (s. 3H, -CH_3_-**10**), 2.57–2.69 (m, 1, -CH-**5**), 5.08–5.14 (m, 1H, -CH = C-**2**), 6.63 (s, 1H, Ph), 6.71 (dd, 1H, *J *= 7.5, 1.8, 1.5 Hz, Ph), 7.05 (d, 1H, *J *= 7.8 Hz, Ph)

### Synergistic effect of simultaneous treatment of xanthorrhizol and curcumin on MDA-MB-231 cells

**Figure 3:**
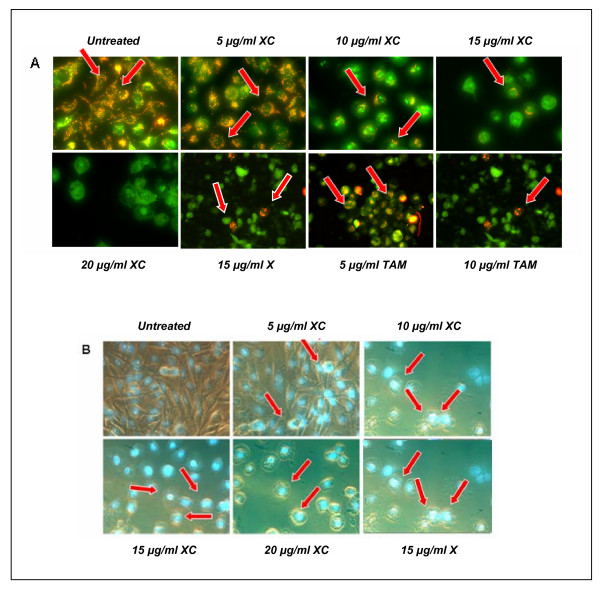
**Isobolograms and combination indexes at 50% effect level of simultaneous and sequential exposure of MDA-MB-231 cells to xanthorrhizol and curcumin**. The dashed line (CI = 1) indicates the alignment of theoretical values of an additive interaction between two substances. Values above the dashed line indicate an antagonistic interaction, and values below indicate synergism interaction. Simultaneous treatment involved concurrent 48 h exposure of MDA-MB-231 cells to xanthorrhizol and curcumin (A and B) whereas sequential treatment was 24 h exposure to xanthorrhizol followed by curcumin for subsequent 24 h (C and D) or *vice versa *(E and F). Simultaneous treatment inhibits the growth of MDA-MB-231 cells more synergistically as compared to the sequential treatment. Results are representative of three independent experiments conducted.

Simultaneous treatment of fixed combination ratio with an increasing fraction of curcumin has showed a synergistic cytotoxic effect in MDA-MB-231 cells as revealed by the isobologram and combination indexes method (Figure 3A, 3B). Synergistic activity was commenced from the combination xanthorrhizol-curcumin 3:7 to 1:9. These data reflects the vital involvement of curcumin in the sensitiviy of MDA-MB-231 cells towards the test agents.

### Cytotoxicity of sequential treatment of xanthorrhizol-curcumin (X-C) and curcumin-xanthorrhizol (C-X) on MDA-MB-231 cells

We had conducted two types of sequential treatments where xanthorrhizol or curcumin was used to treat the MDA-MB-231 cells for 24 hours, followed by the removal of total medium and replenished again with curcumin- or xanthorrhizol-added medium at a fixed combination ratio as in the simultaneous treatment for further 24 hours. The first treatment (X-C) showed antagonism at lower fraction of xanthorrhizol but approaching additivity when the xanthorrhizol was at higher fraction (Figure 3C, D). In contrary, the latter treatment (C-X) resulted in synergistic effect at higher fraction of curcumin and this trend was quite similar with cells that are treated simultaneously with xanthorrhizol and curcumin (Figure 3E, F). Results from both sequential treatments imply the cytotoxicity of the combined treatment of xanthorrhizol and curcumin was schedule-dependent and curcumin may plays a major role in enhancing the cytotoxicity of xanthorrhizol in the MDA-MB-231 cells. Hence, simultaneous treatment was suggested as the better treatment route as compared to both the sequential treatments.

### Disruption of mitochondrial membrane potential of MDA-MB-231 cells after simultaneous treatment with xanthorrhizol-curcumin

The cationic dye MitoCapture™ was able to enter the cell cytoplasm, accumulate and aggregate in mitochondria, thus producing a bright red fluorescence. This red fluorescence was easily distinguished in untreated MDA-MB-231 cells. Remarkably, increasing doses of xanthorrhizol-curcumin simultaneous treatment resulted in significant reduction of this red fluorescence. On the other hand, there were an increasing number of treated cells which only fluoresces green. This is due to the inability of MitoCapture™ to accumulate in mitochondria as the mitochondrial transmembrane potential alters during apoptosis events. They remain as green monomers in the cytoplasm of apoptotic cells. A similar trend of fluorescence was also seen in MDA-MB-231 cells when treated with 15 μg/ml of xanthorrhizol (X) and 5 μg/ml and 10 μg/ml tamoxifen (TAM) respectively (Figure 2A).

### Hoechst 33258 nuclear staining

**Figure 4:**
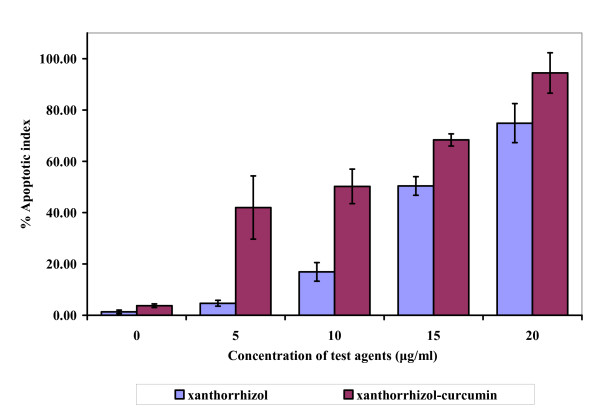
**Apoptosis level after treatment of xanthorrhizol and simultaneous treatment of xanthorrhizol-curcumin as determined from Hoechst 33258 staining**. The treatment for 24 h induced exponential apoptotic cell death in a dose dependent manner as compare to untreated control. Results are presented as means ± SD of 3 independent experiments. *p < 0.05, **p < 0.005 statistically significant values relative to untreated control.

The Hoechst 33258 dye was able to diffuse through intact membranes of MDA-MB-231 cells and stain their DNA. As the concentration of combined xanthorrhizol-curcumin increased, single intense fluorescence and multiple strong fluorescence signals were produced in the cell nuclei (Figure. 2B). The control culture (untreated MDA-MB-231) was uniformly stained with no substantial fluorescence signal, whereas the treated cells showed clear apoptotic morphology. According to the fluorescence and phase contrast images, shrinkage of cells and plasma membrane convolution were all observed in the treated cells. Similar apoptotic morphology was also observed in the MDA-MB-231 cells treated with 15 μg/ml xanthorrhizol (X). The number of apoptotic cells was determined and expressed as percentage of apoptotic index. Simultaneous treatment with combined xanthorrhizol-curcumin resulted in a dose dependent increase in apoptotic cells (Figure 4).

### Treatment with xanthorrhizol-curcumin induced the inter-nucleosomal excision in MDA-MB-231 cells

**Figure 5:**
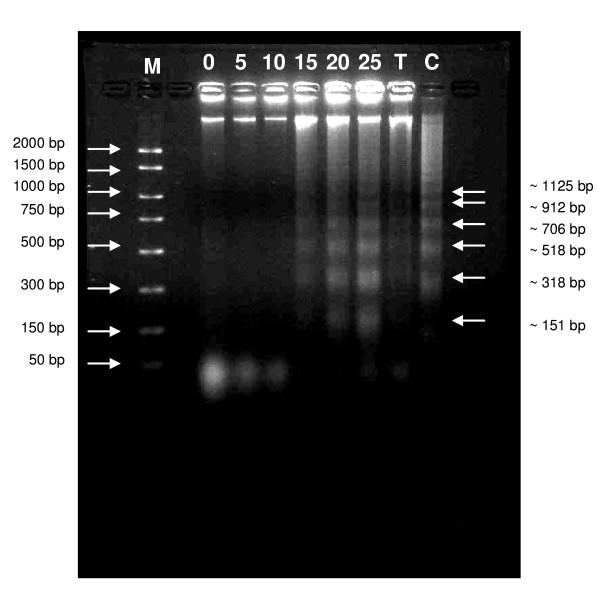
**Simultaneous treatment of xanthorrhizol and curcumin induces DNA fragmentation in MDA-MB-231 cells**. At lower doses of treatment, only high molecular weight intact DNA was observed whereas small fragments of DNA were highlighted at higher doses of treatment. The fragments of DNA have interval molecular weight of ~180 bp, suggesting an apoptotic event. Results are representative of three independent experiments carried out. M – Marker. 0, 5, 10, 15, 20, 25 μg/ml – DNA samples from xanthorrhizol-curcumin (XC)-treated cells at different concentration. T – DNA sample from taxol-treated MDA-MB-231 cells (positive control). C – DNA sample from actinomycin-treated HL 60 cells (positive control).

DNA fragmentation is a biochemical hallmark of apoptotic cell death. From the agarose gel, DNA samples from the treated MDA-MB-231 cells exhibit intact genomic DNA (gDNA) and clear DNA ladders during treatment at higher doses (Figure 5). Treatments at lower doses showed only intact gDNA and do not marked any DNA laddering or even smearing effect. When the multiple bands were analyzed, they showed ~180 bp inter-nucleosomal excision, thus confirming apoptosis but not necrosis had taken place. Lane T and C consist of DNA sample from taxol-treated MDA-MB-231 cells and actinomycin-treated HL 60 cells respectively as positive control. The same pattern of DNA laddering was observed.

## Discussion

Plants have been utilized as medicines for thousands of years. These medicines initially took the form of crude drugs such as powders, tea and other formulations. The use of medicinal plant was then developed into anticancer drugs. This involves the isolation and characterization of pharmacological active compounds. More recently, drug discovery techniques have been moved on to the use of combined active compounds where they are believed to be more active as compared to the single agent itself. Therefore, the efficacy of treatment would increases and the possibility of toxic effect may be lowered due to the extremely low usage of drug [29]. Both xanthorrhizol and curcumin have been reported to have a wide spectrum of pharmacological activities [20,30-35]. They also involved in some anticancer studies [2-7,36,37]. Moreover, curcumin is currently involved in early phase of clinical trial as potential chemopreventive agent [38-42]. Hence, it is rational to investigate the combined effect of xanthorrhizol and curcumin as a new antiproliferative agent for breast cancer cells.

In the present study, the biologically active xanthorrhizol (**1**) was isolated as light brownish essential oil. Analysis of ^1^H-NMR spectrum of 1, revealed that it has a characteristic sesquiterpenoid skeleton, closely related to α-curcumene, a 1-(1,5-dimethyl-hex-4-enyl)-4-methyl-benzene derivative with a bisabolane-type sesquiterpenoid [36,43]. The mass fragmentation of **1 **was very close to that reported by Itokawa and colleagues [36]. The methyl proton attached to the aromatic functional group, next to the hydroxyl group gives resonance at δ 2.238 ppm (s. 3H, -CH_3_-**10**). The existence of phenyl group in **1 **was suggested by the proton resonance at δ 6.630 (s, 1H, Ph), 6.712 (dd, 1H, *J *= 7.5, 1.8, 1.5 Hz, Ph), 7.049 (d, 1H, *J *= 7.8 Hz, Ph) in the ^1^H NMR spectrum. The results consigned are in agreement with a bisabolane sesquiterpeoid skleton in 1. Comparison of the spectroscopic properties with those related compound previously isolated supported the structural assignments [43].

From the isobologram and combination index analysis, simultaneous treatment of xanthorrhizol and curcumin made the best treatment as compare to the sequential treatments. Both simultaneous and sequential treatment of xanthorrhizol and curcumin have highlighted that the involvement of curcumin is vital in the growth inhibition of MDA-MB-231. However, a study on drug and drug interaction, pharmacognosy and other research need to be conducted to confirm their intervention action.

Study on the mode of cell death induced by the simultaneous treatment of combined xanthorrhizol and curcumin was conducted in this study. Apoptosis is a programmed cell death which is activated to expel damaged cells, excessive numbers of cells and cells that are not needed during the development and normal tissue homeostasis [44]. Failing of trigger apoptotic cell death may lead to the development of neoplasia [45]. Therefore, cytotoxicity effect via the induction of apoptosis was considered as criteria for the identification or screening for a new cancer chemotherapy agent [46], and most chemotherapeutic drugs induce apoptosis in cancer cells [47]. The inability of MDA-MB-231 cells to undergo apoptosis in response to anticancer stimuli has been highlighted in recent study [48]. However, our investigations showed that xanthorrhizol was able to induce apoptotic cell death in the MDA-MB-231 cell line. The combination treatment was tested to induce apoptosis cell death. There were few hallmarks of apoptosis observed in our study. The depolarization of mitochondrial occurred in a dose dependent manner and this suggest the release of cytochrome c from the mitochondria intermembrane to the cytosol was being facilitated. Cytochrome *c *is a vital protein in leading the cancer cells to mitochondria-dependent apoptosis through a channel of caspases activation [49-51]. However, the exact mechanism of the mitochondrial membrane permeability and the distribution of cytochrome *c *still remain to be elucidated. In our study, tamoxifen was used as positive control as it has been reported to induce apoptosis in MCF-7 and MDA-MB-231 breast cancer cells *via *the mitochondrial pathway involving the release of cytochrome *c *[52].

There was a marked increase of apoptotic index after treatment with combined xanthorrhizol-curcumin. Immunofluorescence images showed that the dying cells exhibit ultrastructural and biochemical features that characterized apoptosis, as shown by the loss of cell viability, DNA condensation, DNA fragmentation and cell shrinkage [3,47,53]. Recently, xanthorrhizol treatment has been reported to cause apoptotic cell death in MCF-7 [3], HeLa [2], and HepG2 cancer cell lines as well [4]. Cell shrinkage happens only in apoptotic cell death. Cells intracellular concentration of monovalen ions (K^+ ^and Na^+^) was able to inhibit activation of cell death cascade. Therefore, the ions will be expelled out during apoptotic event and this will cause the cell to shrink. As compare to necrosis, ionic homeostasis occurs due to the drastically decrease of energy level. This event will cause the increase of cell volume and subsequently, swelling and total cell lysis will be observed [54,55].

DNA laddering is a definitive indication of whether cells are undergoing apoptosis or necrosis [56]. Inter-nucleosomal excision at ~180 bp interval was observed after the treatment with xanthorrhizol-curcumin. This DNA fragmentation event occurred in-line with the increased level of apoptotic index. It also indicates the point of no return for a dying cell as cleavage of gDNA by endogenous endonucleases during apoptosis is an irreversible event that commits cell to die [57]. Hence, the possibility of cells regain the proliferative activity is very low and thus, simultaneous treatment of xanthorrhizol-curcumin has advantage to become a new combination treatment for MDA-MB-231 human breast cancer cells.

## Conclusion

The experimental evidence from this study suggests that simultaneous treatment of xanthorrhizol and curcumin exhibits synergistic effect towards human breast carcinoma cells MDA-MB-231 whereas sequential treatment with xanthorrhizol and curcumin or *vice versa *did not showed greater advantages as compare to the simultaneous treatment. The combination treatment induced apoptotic cell death as the experimental evidence from this study revealed that alteration of membrane potential, DNA condensation, cell shrinkage and DNA fragmentation were observed after the treatment. Taken together with the antiproliferative activity of xanthorrhizol and curcumin, antimetastatic activity of xanthorrhizol, organ protective effect of xanthorrhizol, we believe combined treatment of xanthorrhizol-curcumin could be a potential antiproliferative agent for invasive breast cancer cells. However, in-depth studies especially in the molecular mechanism of apoptotic cell death need to be conducted to assure its efficacy.

## Methods

### Extraction and isolation of xanthorrhizol

Fresh rhizomes of *C. xanthorrhizza *Roxb. were chopped and essential oils were obtained by hydrodistillation in a modified Clevenger-type apparatus. Distilates were then extracted 3 times with diethyl ether. The pooled organic phases were dried and subjected to two rounds of silica gel vacuum column chromatography (VCC) separation (Merck silica gel 60, 0.040–0.063 mm; and Merck silica gel 60, 0.063–0.200 mm respectively). The columns were eluted with petroleum-ether with gradual increase ratio of diethyl ether. The existence of xanthorrhizol in each fraction was pre-identified using thin layer chromatography (TLC) and gas chromatography-mass spectrometry (GC-MS) by comparing the retention factor (R_f_) in TLC profile and the total ion chromatogram (TIC)/Mass spectra in GC-MS profile respectively with those of authentic reference substance (Alexis Biochemicals, Switzerland). Fractions were subjected to GC-MS analysis on an Agilent system with a model 6890 gas chromatography, a model 5073 Mass Selective detector (MSD) and an Agilent Chemstation data system. The following GC oven temperature program was used: 40°C initial temperature; held for 2 min; increased at 5°c/min to 230°C with split (50:1) injection.

**Scheme 1:**
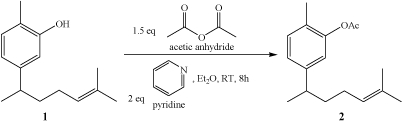
(Acetylation process)

**Scheme 2:**
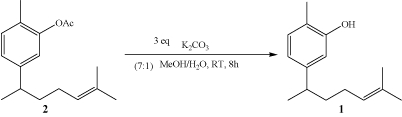
(Hydrolysis process)

From our previous study, xanthorrhizol is not able to be separated by normal CC using mixture of solvents with different polarity as other compounds always appeared together as co-eluents. In an effort to purify xanthorrhizol from the final fraction from VCC, subsequent reaction steps (Scheme 1 and Scheme 2) were conducted to facilitate the isolation and purification. Xanthorrhizol was reported as a potential chiral starting material for the synthesis of other sesquiterpenoids [43]. As a result, we started the process of acetylation towards fractions that comprise compound **1 **and other co-eluents to produce a lower polarity compound **2 **namely, acetyl-xanthorrhizol (Scheme 1).

This particular fraction which consists of compound **2 **and other components was subjected to open CC to separate compound **2 **and other compounds. Compound **2 **was easily separated from other compounds in this step as its polarity was increased after the acetylation process. Following fractions which contain only **2 **were then pooled and undergone hydrolysis to produce back xanthorrhizol (**1**), as showed in scheme 2. Subsequent partition was then able to separate **1 **into organic phases in EtOAc. Finally, evaporation of the organic solvent yielded **1 **as a light brownish oil with 71% yield.

Identification of **1 **was carried out by comparing its GC mass spectral to the authentic reference substance (Alexis Biochemicals, Switzerland), referring to the NIST98 database/Chemstation data system (National Institute of Standards and Technology, Gaithersburg, MD, USA). Subsequent identification was conducted by using ^1^H-NMR analysis. NMR spectral (300 MHz) of **1 **was recorded on a Bruker Avance 300 Spectrometer using CDCl_3 _as solvent. Chemical shifts are reported in ppm with reference to residual solvent [CHCl_3 _(7.26)].

### Cell culture

MDA-MB-231 human breast cancer cell line was obtained from the American Type Culture Collection (ATCC, Manassas, VA, USA). They are maintained in Dulbecco's modified Eagle's medium (DMEM; Invitrogen Co., Carlsbad, CA, USA) supplemented with 5% heat-inactivated fetal calf serum (Invitrogen Co.), 100 U/ml penicillin and 100 mg/ml streptomycin (Flowlab, Sdyney, Australia) in a humidified atmosphere of 5% CO_2 _in air at 37°C. Cells were kept in the logarithmic growth phase by routine passage every 2–3 days using 0.025% trypsin-EDTA treatment.

### Cell growth assay

The antiproliferative activity of fixed ratio combination of xanthorrhizol and curcumin (from 1:9 to 9:1) towards MDA-MB-231 human breast cancer cell line was evaluated by using the sulforhodamine B (SRB) method, as previously described [58,59]. Cells were seeded 24 hours prior to treatment in a 96-well plate at plating densities 15, 000 cells/well in order to obtain semi-confluent cultures. One plate from the cell line was fixed in situ with TCA (Sigma Chemical Co.), to represent a measurement of the cell population for each cell line at the time of drug addition (*T*_*z*_). Combination solutions were dissolved in DMSO (Sigma Chemical Co.) and followed by a 2× serial dilution for 6 points ranged from 40 μg/ml to 1.25 μg/ml. The final concentration of DMSO used in the corresponding wells did not exceed 1% (v/v), which affect cell viability. Following drug addition, the plates were incubated for an additional 48 hours.

At the end of incubation, cells were fixed in situ with 50 μl of cold 50% (w/v) TCA and incubated for 1 hour at 4°C. The plates were then washed with tap water and air dried. SRB (Sigma Chemical Co.) solution (100 μl) at 0.4% (w/v) in 1% acetic acid was added to the corresponding wells and further incubated for 10 minutes at room temperature. After staining, unbound dye was washed out by 1% acetic acid and the bound stain was subsequently solubilised with 10 mM trizma base (Sigma Chemical Co.). The absorbance at 505 nm was read on a spectrophotometric plate reader.

Dose-response curves were constructed to obtain the response parameter which was the GI_50_. Percentage growth is calculated as: [(*T*_*i *_- *T*_*z*_)/(*C *- *T*_*z*_)] × 100 where (*T*_*z*_) is time zero, (*C*) is control growth and (*T*_*i*_) is test growth in the presence of drug at the six concentration levels. The GI_50 _value (growth inhibitory activity) corresponds to the concentration of test agents causing 50% decrease in net cell growth which was calculated from [(*T*_*i *_- *T*_*z*_)/(*C *- *T*_*z*_)] × 100 = 50. All data were derived from 3 independent experiments.

### Isobologram and combination-index analysis

Drug synergy was determined by the isobologram analysis and combination-index (CI) methods, as described elsewhere [60,61]. Isobologram analysis was used to determine the effects of combinations of drugs on MDA-MB-231 cells whereas the CI method is a mathematical and quantitative representation of two-drug pharmacologic interaction. Using data from the growth inhibition assay, classic isobologram was constructed and CI values were then calculated by using the following formula: CI = (*d*1/*D*_*x*_1) + (*d*2/*D*_*x*_2), where *D*_x _is the dose of one drug alone required to produce a 50% growth inhibition, and *d*1 and *d*2 are the doses of xanthorrhizol and curcumin respectively, in combination that produce the same effect (50% growth inhibition). A CI of > 1 implies antagonism, = 1 is additivity, and < 1 is synergy.

### Analysis of alteration of mitochondrial membrane potential

Changes in the mitochondrial membrane potential of treated MDA-MB-231 cells were examined using a fluorescent microscope (Imaging Source Europe GmbH) and MitoCapture™ Mitochondrial Apoptosis Detection Kit (BioVision Research, Moutain View, CA, USA), according to the manufacturer's instructions. Briefly, treated and untreated cells were washed with PBS. One μl of MitoCapture dye was diluted to 1 ml pre-warmed incubation buffer immediately prior to use. The cells were then incubated in 500 μl of the diluted MitoCapture solution at 37°C for 20 minutes in a CO_2 _incubator before being observed under a fluorescence microscope using a band-pass filter.

### Hoechst 33258 nuclear staining assay

Nuclear staining with Hoechst 33258 (Sigma Chemical Co.) was performed as described elsewhere [3,62]. Briefly, treated and untreated cells were washed with phosphate buffered saline (PBS) followed by incubation in 10 μM Hoechst 33258 solution at room temperature for 20 min. At the end of incubation, cells were washed extensively with PBS. Nuclear morphology was then examined under a fluorescence microscope (Imaging Source Europe GmbH, Bremen, Germany). To quantify the apoptotic index, the percentage of single and multi intense-fluoresced cells (apoptotic morphology) were calculated from five random microscopic fields at ×40 magnification.

### DNA fragmentation assay

DNA fragmentation assay is a definitive assay to confirm apoptotic cell death has taken place in the cultures. DNA fragmentation assay was conducted using the Suicide Track™ DNA Ladder Isolation Kit (Calbiochem), according to manufacturer's instructions. Briefly, MDA-MB-231 cells were cultured in a T-25 flask and treated with combined solution of xanthorrhizol-curcumin for 24 hours. Floating and and trypsinized-adherent of treated and untreated cells were collected by centrifuging at 900 × g for 5 min. Cell pellets were then resuspended in 250 μl of extraction buffer following incubation on 4°C for 30 min. The suspensions were then centrifuged at 16, 000 × g for 5 min at room temperature. The resulting supernatant were transferred to a clean tube, followed by the addition of 10 μl of RNase A solution and incubated at 37°C for 1 h. DNA isolation buffer was added and further incubated at 50°C for overnight. During the DNA precipitation step, 2 μl of Pellet Paint^® ^Co-precipitant was added to the suspension together with 30 μl of 3 M sodium acetate and 311 μl of 2-propanol. Samples were mixed by inversion and then centrifuged at 16, 000 × g for 5 min. The resulting pellets were washed with 70% and 100% ethanol. The DNA samples were then air-dried and resuspended in 50 μl of resuspension buffer (10 mM Tris, 1 mM EDTA). Finally, DNA samples were separated in 2% agarose gel in 1× TAE. Positive control was supplied by Calbiochem which consist of 1 × 10^6 ^HL60 cells treated with 0.5 mg/ml Actinomycin D for 19 h.

### Statistical analysis

All data were expressed as mean ± standard deviation. The statistical differences were analyzed using one-way ANOVA followed by a Tukey Honestly Significantly Different (HSD) test. Values of *p *< 0.05 were considered significant.

## Competing interests

The authors declare that they have no competing interests.

## Authors' contributions

All authors read and approved the final manuscript.

YHC was the PI at Institute for Medical Research and was responsible for study design, interpretation of the data, preparation and revision of the manuscript. RS, HMS, and BAAD coordinated the extraction of xanthorrhizol. FJN and TTT contributed to the *in vitro *cell culture, SRB, and immunostaining experiments. HLPA and NRA contributed to the experimental design and supervision. ZI coordinated the study and the experiment consult.
